# News and Miscellany

**Published:** 1903-10

**Authors:** 


### News and Miscellany.

There is hope for the physician who will admit that he is guilty of malpractice every day of his professional life.

Sciatica.—Kuhn states that deep injections of antipyrin, at a point midway between the great trochanter and the tuberosity of the ischium, will relieve the pain of sciatica.—*Med. Rec.*

Consumption.—It can not be too strongly insisted upon that all who are prone to this disease must be kept as much as possible in the open air. Even at home this may be done by keeping all windows of sleeping rooms open to their fullest extent, using proper clothing to preserve heat, and during the daytime keeping the patient out of doors, protected from the weather, no matter what the circumstances of the patient are. Constantly we find such people confined to a close room, as if the pure air were dangerous to breathe. Added to this the want of abundance of nourishing food, and it is not to be wondered at that the disease rapidly carries off the patient. In many instances, the moment the patient shows what is the nature of the ailment, we find him scrupulously secluded to his room. The air, already foul, made more so by the presence of visitors, filth, tobacco, etc., thus preventing the lungs from being aided in their efforts to throw off the disease, in fact, increasing the power of the poison by furnishing it a soil to grow in. Disinfection, cleanliness, fresh air—all aid in preventing the progress of the disease and prolong life, if not curing the case.—*Public Health.*

Use of Normal Salt Solution.—Salt solution is employed in all diseased conditions associated with either hemorrhage or intense toxemia. In hemorrhage it replaces the fluid lost to the tissues and refills the blood vessels, thereby giving the heart something on which to work. It stimulates the cardiac ganglia; sustains the nutrition of the heart itself, rendering it possible for the remaining blood to be propelled to the vital centers; and sustains life temporarily until new blood can be formed. It raises the temperature to normal and relieves collapse. In toxic conditions it excites diaphoresis and diuresis, lowers the specific gravity of the urine, increases phagocytosis, dilutes the poisons circulating in the bloodstream, and by a process of cell-lavage removes the toxin from the paralyzed cell, allowing it to resume its normal function.—*H. F. Thompson, in Med. News.*

DOCTOR (reclining in his easy chair by the fire, in his soft *robe de chamber,* reading the TEXAS MEDICAL JOURNAL; his pretty wife is waiting for him to get through with it, so she can read it—children all asleep)—"Dr. Daniel gets out a most interesting journal; it is the most intensely original journal published. It takes money to do it. I do not know how I could get along without it, and yet—by jingo! I haven't paid my subscription, notwithstanding the doctor has politely reminded me, several times. I must go tomorrow and send him a postal note. I've had pretty good luck collecting."

Pretty Wife—"Do, don't forget it, my dear; here, let me tie a string around your finger as a reminder. Remember—'Do unto others as you know how it is yourself'—'the rolling hen never gets moss,' or words to that effect."

Doctor—"I'll *do* it, sure! That is the man I am of a kind." (Exit doctor for the postoffice.)

---

THE RESULTS OF THE TREATMENT OF FOUR HUNDRED AND EIGHT CASES OF TYPHOID FEVER, WITH A REPORT OF EXPERIMENTS WITH A NEW METHOD.—H. C. McCormick states that the local application of guaiacol for the reduction of temperature has proved so effective and safe a remedy, that after having applied it 3,150 times, he heartily recommends it. The right iliac region is cleansed and dried, and alcohol applied. From five to ten drops of guaiacol are slowly dropped on the surface and rubbed in with the hand from ten to fifteen minutes. The part is then covered with oiled paper. The antipyretic effect lasts from two to four hours. The author treated four patients with acid sulphate of sodium. They were admitted on or before the seventh day. The temperature charts showed, from the time they came under the influence of the sodium salt, a fall of about one degree, and the morning remission was almost entirely abolished. The author finds that in a solution of 1 to 300 the acid sulphate of sodium is germicidal to typhoid bacilli, increases leucocytosis, and thus helps nature to combat the disease, neutralizes typhotoxin, and in a measure lessens delirium and other symptoms attributed to this poison, prevents, to a great degree, the congestion of Peyer's patches, and by this means prevents to some extent hemorrhage and perforation. It will resist bacilli or toxin in any part of the body, and greatly lessens the complications of the disease. It is a prophylactic, and is not neutralized in the stomach. It replaces physiologically the deficient hydrochloric acid. It furnishes an abortive treatment. It may be used to purify contaminated drinking water by allowing the solution to stand from twelve to fifteen minutes before being taken.—*The Theraputic Gazette.*

---

IT IS NOW DEFINITELY SETTLED that strabismus is *not* caused by *cocci.* Bangs says the cases of cock eye *he* sees have been produced by spasmodic and frantic efforts to *see the point* of some of the "Red-Back's" jokes.

THE DOCTOR'S RECREATION SERIES.—12 vols., octavo. The Saalfield Publishing Company, Cleveland, Ohio, will very shortly issue twelve volumes of books under the above title. They are not, strictly speaking, medical books, but they are, nevertheless, books that all doctors will enjoy reading—for "recreation." Among them (Vov. VI) is "Passages from the Diary of a Late Physician," by Samuel Warren, author of the immortal story, "Ten Thousand a Year." I have received a letter from Dr. S. W. Kelley, late editor of the Cleveland *Medical Gazette,* who is managing this business, saying these books will be sent the "Red-Back" for review, soon. Then I'll tell you more about them. I'll bet they are good.

---

SUBSCRIPTION BILLS have been sent to all my subscribers. I will appreciate prompt remittance. Add 10 cents if personal check is sent. Costs me 10 cents for each $1 check. See?

---

MISSISSIPPI has at last established a medical college. The Medical Department of the University of Mississippi began its first session September 17th. It is located at Oxford, I presume, the seat of the university. Our Mississippi contemporary, in an editorial on the subject, is a little nebulous. He leaves readers in the dark as to location, faculty and other important details. On account of lack of hospital facilities, it is to be presumed, the new school will not attempt clinical instruction, but will give only the first two years course, teaching thoroughly "the so-called scientific branches of medicine," says the Mississippi *Medical Record.*

---

ANOTHER LANDMARK GONE.—Dr. J. Y. Bradfield, retired physician and banker, of Daingerfield, Tex., died at his home October 5th, aged 68 years. He was a graduate of the Medical Department University of Georgia, class 1858.

---

RHEUMATIC DYSMENORRHEA.—

| | |
|---|---|
| Am. Hydrochlor | 2½ ozs. |
| Tr. Stramonii | ½ oz. |
| Tr. Cimicifugæ | 1 oz. |
| Syr. Glycyrrhizæ | 2 drms. |
| Tongaline, q. s. ad | 6 ozs. |

M. Sig.—Teaspoonful three times a day.

---

NEW ORLEANS POLYCLINIC.—Seventeenth annual session opens November 2, 1903, and closes May 28, 1904. Physicians will find the Polyclinic an excellent means for posting themselves upon modern progress in all branches of medicine and surgery. The specialties are fully taught, including laboratory work. For further information, address New Orleans Polyclinic, postoffice box 797, New Orleans, La.

---

"COSTIC."—The St. Joseph *Medical Herald* says the "Red-Back" is "too costic," whatever that may be.

For Sale.—In small town, four room cottage, wind mill and outbuildings; office on separate lot; $3000 practice thrown in; collection 90 per cent. No opposition. Price $600. U. E. G. Dyer, M. D., Star, Tex.

Will Repair Your Electrical Machines.—Static and all electrical medical apparatus put in running order. I am also agent for electrical and X-ray apparatus. Oliver Brush, 710 Colorado Street, Austin, Texas.

Dr. G. Frank Lydston, of Chicago, will shortly publish a work on sociology, entitled "The Diseases of Society," which will embrace the crime question, the social evil, etc.

Attention is called to Mrs. Hagerty's card, "Deep-breathing and Physical Culture." The editor personally endorses and recommends Mrs. Hagerty to the confidence and patronage of the readers of the Journal.

The American Congress of Tuberculosis will meet in St. Louis, October 3, 4 and 5, 1903. It has been incorporated into the program of congresses to be held there during the great exposition, and the American government has taken a hand in it officially. It will become an International Congress. Acting Secretary of State Adee writes Hon. Clark Bell, Chairman of the Executive Committee (September 21, 1903), informing him that instructions have been sent by that department to the diplomatic officers of the United States, accredited to the Central and South American States, Mexico, Hayti and San Domingo, and also to our ambassadors at London and Paris, and ministers at the Hague and Copenhagen, with regard to the British, French, Dutch and Danish colonial governments, to invite all these governments to send representatives to the great congress at St. Louis. It is believed that these instructions will result in a full representation by all American States and our colonial governments, as well as from other governments, and Secretary Adee expresses the hope that it will be so.

Swallowed an Elevator.—Farmer Peachstone: Gosh a mighty, doctor, can't you do sumthin' for a feller more'n you're doin'? I'm plumb tired of six meals a day, three goin' up and three goin' down."—*Ex.*

A Point in Differential Diagnosis.—Mrs. McGinnis: "Mrs. Murphy, dear, how do you distinguish them darling twins of yours, the one from the other?"

Mrs. Murphy: "I'ts dead easy; I just puts my finger in Dennis's mouth, and if he bites,—I known it's Mike!"

